# The ethics of facial recognition technologies, surveillance, and accountability in an age of artificial intelligence: a comparative analysis of US, EU, and UK regulatory frameworks

**DOI:** 10.1007/s43681-021-00077-w

**Published:** 2021-07-29

**Authors:** Denise Almeida, Konstantin Shmarko, Elizabeth Lomas

**Affiliations:** 1 Department of Information Studies, UCL, London, UK; 2 Department of Economics, UCL, London, UK

**Keywords:** Facial recognition technology, Accountability, AI ethics, AI regulation, Data protection, GDPR, Human rights, Impact assessment, Law enforcement, Privacy, Surveillance

## Abstract

The rapid development of facial recognition technologies (FRT) has led to complex ethical choices in terms of balancing individual privacy rights versus delivering societal safety. Within this space, increasingly commonplace use of these technologies by law enforcement agencies has presented a particular lens for probing this complex landscape, its application, and the acceptable extent of citizen surveillance. This analysis focuses on the regulatory contexts and recent case law in the United States (USA), United Kingdom (UK), and European Union (EU) in terms of the use and misuse of FRT by law enforcement agencies. In the case of the USA, it is one of the main global regions in which the technology is being rapidly evolved, and yet, it has a patchwork of legislation with less emphasis on data protection and privacy. Within the context of the EU and the UK, there has been a critical focus on the development of accountability requirements particularly when considered in the context of the EU’s General Data Protection Regulation (GDPR) and the legal focus on Privacy by Design (PbD). However, globally, there is no standardised human rights framework and regulatory requirements that can be easily applied to FRT rollout. This article contains a discursive discussion considering the complexity of the ethical and regulatory dimensions at play in these spaces including considering data protection and human rights frameworks. It concludes that data protection impact assessments (DPIA) and human rights impact assessments together with greater transparency, regulation, audit and explanation of FRT use, and application in individual contexts would improve FRT deployments. In addition, it sets out ten critical questions which it suggests need to be answered for the successful development and deployment of FRT and AI more broadly. It is suggested that these should be answered by lawmakers, policy makers, AI developers, and adopters.

## Introduction

Law enforcement agencies globally are constantly seeking new technologies to better ensure successful detection and prosecution of crimes to keep citizens and society safe. In addition, there is a public expectation to deliver value for money and where possible to provide economic efficiencies and reduced labor costs, which potentially new technologies can help deliver. Over the last decade, many new technologies have been harnessed by law enforcement agencies including, but not limited to surveillance cameras, automated license plate readers, body cameras, drones, and now facial recognition technologies (FRT). Law enforcement agencies have been at the forefront of FRT adoption due to the benefits that can be seen to be derived and justified in this space. However, each of these technologies changes the relationships between law enforcement operatives and citizens and requires the negotiation of new boundaries and revised accountability requirements. It is important to recognise that each technology has encroached on citizens’ privacy and relationship with the state. As such, what is being deemed as acceptable in terms of reshaping boundaries is under scrutiny and debate. However, the decisions being made in regard to technology adoption are not currently uniform. There are distinct differences in technology adoption and roll out nation to nation and in some national contexts state to state. These largely depend on the legal landscape in terms of privacy/data protection legislation and citizen acceptance and expectations of surveillance. Within this context, COVID-19 has further pushed the boundaries of privacy, with nations introducing new measures to track citizens’ movements and connections to contain the spread of the virus. However, the shift in enhanced monitoring, surveillance and privacy disclosures, and accountability in this regard is being questioned globally, drawing attention to changes and challenges [1, 2]. This latter question of accountability and acceptable privacy limits is critical in terms of balancing rights and responsibilities for FRT.

Accountability provides for the obligation to explain, justify, and take responsibility for actions. In the context of the state and law enforcement, the state is obligated to be responsible for and answer for the choices it makes in terms of the technologies it rolls out and how these impact in particular case contexts. Many questions about the use of FRT and Artificial Intelligence (AI) have yet to be fully resolved. FRT usage by law enforcement agencies provides a strong case study for considering aspects of FRT and AI ethics more generally. It provides for a very understandable use of personal data with clear impacts on individuals rights.

This article considers the complexity of the ethical and regulatory dimensions at play in the space of FRT and law enforcement. The paper starts by providing a brief explanation of FRT, followed by an analysis of the use of FRT by law enforcement and legal approaches to the regulation of FRT in the US, EU, and UK. We conclude by recommending that there must be better checks and balances for individuals and societal needs. There needs to be accountability through greater transparency, regulation, audit and explanation of FRT use and application in individual contexts. One critical tool for this is the impact assessment, which can be used to undertake data protection impact assessments (DPIA) and human rights impact assessments. Ten critical ethical questions are framed that need to be considered for the ethical development, procurement, rollout, and use of FRT for law enforcement purposes. It is worth stating these from the outset:

1. Who should control the development, purchase, and testing of FRT systems ensuring the proper management and processes to challenge bias?
2. For what purposes and in what contexts is it acceptable to use FRT to capture individuals’ images?
3. What specific consents, notices and checks and balances should be in place for fairness and transparency for these purposes?
4. On what basis should facial data banks be built and used in relation to which purposes?
5. What specific consents, notices and checks and balances should be in place for fairness and transparency for data bank accrual and use and what should not be allowable in terms of data scraping, etc.?
6. What are the limitations of FRT performance capabilities for different purposes taking into consideration the design context?
7. What accountability should be in place for different usages?
8. How can this accountability be explicitly exercised, explained and audited for a range of stakeholder needs?
9. How are complaint and challenge processes enabled and afforded to all?
10. Can counter-AI initiatives be conducted to challenge and test law enforcement and audit systems?

Finally, it should be established that while law enforcement agencies are at the forefront of FRT adoption, others can learn valuable ethical lessons from the frameworks put in place to safeguard citizens’ rights and ensure accountability through time. Many of these same questions are applicable to AI development more broadly and should be considered by law makers to legislate and mandate for robust AI frameworks.

## Facial recognition technologies (FRT)

Facial recognition in essence works by capturing an individual’s image and then identifying that person through analysing and mapping of those captured features comparing them to identified likenesses. Facial images, and their careful analysis, have been a critical toolkit of law enforcement agencies since the nineteenth century. However, in the twenty-first century, the application of facial recognition, moving from manual techniques to facial recognition technologies (FRT), to automatically extract and compare features and every nuance of their measurement through the application of artificial intelligence (AI) and algorithms has significantly enhanced this basic tool [3]. As such, the face can be mapped and compared to other data which offers a more formal match and identification to an individual. This can sometimes involve the introduction of other biometric data such as eye recognition data. One-to-one matching provides for certain identification of an individual in a specific context. However, using an identified image in connection with other data banks or data lakes enables one-to-many possibilities and connotations of usage. Matching that can process data at scale presents new possibilities and complexities when considering machine learning, algorithms, and AI.

The context of the situation of FRT rollout and data gathering is potentially all important in terms of how it aligns with citizens’ security versus privacy concerns in differing situations. In 2008, Lenovo launched a new series of laptops that instead of requiring a password, could recognise the face of their authorised user [4]. This functionality was seen as a marketing benefit for Lenovo and clearly users consented and engaged with the capture and use for their own personal computing needs and one-to-one matching. However, there will be distinctions between expectations in one-to-one matching in a more private controlled space for transparent individual benefits versus taking and using a verification process in broader and potentially big data contexts. As the proposed EU regulation on AI suggests, the use of FRT in public spaces is ethically (and legally) significantly different than its use for device unlocking. Citizens will have different expectations about spaces in which surveillance and FRT should be in place. For example, when crossing national border jurisdictions, there has always been an exchange of data and careful identification of individuals and as such FRT may be deemed to be more acceptable in this space as opposed to when moving around public spaces more generally, functioning in working spaces and finally residing within private home dwellings. In each of these spaces, the expectations for active law enforcement and surveillance clearly differ and there are a number of ethical questions to be answered for a successful rollout in different contexts and for different law enforcement purposes. In addition, there are differences between expectations for localised enforcement agencies such as police services and national intelligence agencies undertaking more covert security operations. In each citizen space, and dependent upon the form of law enforcement, there will be different perspectives and concerns from individuals and groups of stakeholders. As such, reaching a consensus in technological rollouts will be a journey. Even in the example of border controls, where ID data have always been exchanged, studies have shown that the views of travellers on acceptable technologies differ from the views of board control guards [5].

In regard to law enforcement, some scholars have advanced the theory that monitoring of social media by law enforcement could be perceived as a ‘digital stop and frisk’, potentially delivering, “everyday racism in social media policing as an emerging framework for conceptualizing how various forms of racism affect social media policing strategies” [6]. This statement evidences concerns about the bias and credibility of law enforcement agencies. Applying this same conceptual framework to, sometimes flawed, facial recognition algorithms without taking accountability for the consequences of this usage could not only lead to further discrimination and victimisation of specific communities, but also to an even greater loss of trust between the general population and law enforcement agencies. In recent years, we have seen an exponential increase in research focused on issues of algorithmic accountability,^1^ with the overarching message being that algorithms tend to reflect the biases of those who build them, and the data used to train them. The extent to which they can be relied on without human checks is one of constant concern, particularly as the use of these technologies as well as identifying individuals is extending their reach to make further judgements about individuals including in regard to their behaviours, motivations, emotions, and protected characteristics such as gender or sexuality [7].

In the specific case of FRT, it is important to understand some aspects at play in the design and roll out that have led to concerns over biases and unbalanced power structures. The majority of technology workers in the West are claimed to be white men, which as such unintentionally influences the development of technologies such as FRT [8]. Input bias has been known about for decades, but has not been fully surfaced in an FRT context. If FRT are trained on white male faces, then there will be implications when it is used to process data related to non-white and female faces. As such, studies have indicated that identification and bias failings do occur [9]. Even where inputs are adjusted, systems can be biased by attempting to meet the anticipated needs of purchasers and users which may skew the system particularly as algorithms are applied and developed through time. In each of these instances, a high proportion of the stakeholders with power and influence are likely to be male and white [10]. These biases can lead to severe consequences, particularly when carried into uses by law enforcement. This brings to the surface issues of power dynamics and citizen trust of its law enforcement.

However, it is equally to be noted that AI has the potential to challenge biases and to be used in innovative ways that can alter existing power dynamics. A significant example of this, is the recent use of FRT by human rights activists and protesters as a way to identify, and hold accountable, law enforcement officers who might be abusing their power [11]. This ‘turn of the tables’ adds a further layer of complexity to discussions of accountability and power. However, while a group of people who typically do not hold power may in limited circumstances use FRT to hold law enforcement accountable, that does not make the technology ethically viable. However, this power shift, if more formally supported, might provide a part of the solution to FRT deployment and its impacts. For example, as images are captured and significant in legal case contexts, AI has the power to potentially assist with identifying deep fakes and calling out adaptions to footage and photographs. As such, it is important to drill down into the use of FRT and the frameworks which sit around FRT.

## The EU and UK legislative landscape for FRT in a law enforcement context

There are currently no FRT specific pieces of legislation in the EU and UK domains, but there are other pieces of legislation that dictate the management and rollout of FRT. In terms of personal data management, the EU’s GDPR, which came into force in 2018 covering all the Member States of the EU, has been seen as setting the bar at the highest level for the management of personal data. As such, for many tech companies operating at a global level, it has been seen as the de facto standard to roll out across all global operations. It is to be noted that as the GDPR came into force, while the UK was part of the EU, it was enshrined into UK domestic legislation and still continues to apply within a UK context. The UK’s ongoing adequacy in terms of alignment to EU GDPR will continue to be judged by the EU.

The GDPR has required systems to be implemented where ‘privacy by design’ (PbD) and ‘privacy by default’ are inbuilt for any personal data processing. Processing covers any activity with personal data including creating, receiving, sharing, and even destroying/deleting personal data. There must be a clear lawful basis for personal data processing, and in addition, the data must be processed fairly and transparently. Within this context, it is important to understand that this does not prevent personal data collection, but does require carefully documented processes and active personal data management through time. In addition, it must be noted that what is considered fair and lawful is potentially open to interpretation and legal debate and contest. In certain instances, consent for processing is required. In addition, there are specific data subject rights such as the right to know what is held on/about you, subject to certain exemptions and to ask for data to be rectified or deleted (the right to be forgotten) in certain circumstances.

Where special category personal data are processed, stricter controls are required. Of note in this regard is biometric data which is categorised as physical or behavioural characteristics that uniquely identify an individual, including but not limited to DNA, fingerprints, faces, and voice patterns as examples. As such FRT are caught under this definition and within Article 9 of the GDPR, it is clarified that biometric data should not be used to identify a person unless an individual has provided explicit consent or alternatively other exemptions exist. One such example of an exempted area across the EU and UK is law enforcement. In the GDPR, personal data management for law enforcement purposes was derogated in Article 23, for determination at Member State level. There is therefore some divergence in terms of how the checks and balances exist between personal data rights and law enforcement rights. Within most EU Member States there is an expectation that for the purposes of pursuing law enforcement to identify and track offenders certain exemptions would exist, and consent would not be required. Within this space, the new technological landscape is further continuing to evolve and as such its rollout and use by law enforcement agencies is not consistent across the EU.

Regardless of certain consent exemptions, other GDPR requirements do still apply, such as PbD, which does provide a framework of accountability for law enforcement. For FRT purposes, a DPIA must be undertaken as a way of demonstrating and achieving PbD. The DPIA is a process of identifying risks that arise from data processing and is mandatory for high-risk applications, such as facial recognition in law enforcement use.^2^ This requires that all aspects of a process are reviewed and considered to ensure that there are justifications for the process; this ensures it is ‘fair and lawful’, it is appropriately targeted, implemented and managed through time. This procedure is not only useful for the FRT operators, as it forces them to scrutinise their algorithms, focus and security, but can also benefit the general public, as, if published, a DPIA can explain data processing in terms that are accessible to any individual, not just an IT specialist. Mandatory publication of the DPIA does not exist, but there is a requirement to be transparent about DP processing and to have in place privacy notices for this reason.

Another important GDPR requirement is the need to have a Data Protection Officer (DPO) within any public authority or private entities where the core activities require large scale, regular, and systematic monitoring of individuals or large-scale processing of special category data or data relating to criminal convictions or offences. As such, this does mean that law enforcement agencies and businesses providing processing services will be required to have a DPO. The DPO is required to advise an organisation on its data protection compliance. In addition, were an organisation to fail to fully comply with the GDPR, the DPO would act as a whistle-blower reporting to the relevant national ombudsman on data protection.

Each EU Member State and the UK has a regulatory requirement which establishes an oversight, complaint, and investigatory regime to be in place, a data protection ombudsman/regulator. There are currently 27 data protection authorities in the EU, one for each country, plus the European Data Protection Supervisor, which oversees EU institutions and bodies. The UK also has a data protection supervisor. The exact responsibilities of the organisations differ, but all of them are tasked with monitoring and ensuring data protection and privacy compliance regionally on behalf of their citizens. In accordance with this mandate, it is not uncommon to see these authorities actively intervening in relevant disputes, sometimes even before any citizen complaints are filed. The benefit to accountability of these organisations is obvious—the data protection regulators have bigger budgets and better legal teams than most individuals, meaning that they are more effective in holding FRT operators accountable. The authorities with enforcement powers can bypass litigation entirely, issuing fines and orders faster than a court would be able to. These factors ensure that the FRT providers and operators should never get complacent.

Separately, citizens may bring forward lawsuits for data protection failings, but the ability to complain to a regulator provides the citizen with a cheaper alternative and one which should actively investigate and oversee any organisational data protection failings. The regulators are publicly funded and the resources for each across the EU and UK vary significantly. The extent of investigations and the timeliness of dealing with complaints have both been areas of criticism. For example, in 2020, a group of cross-party Members of the UK Parliament wrote complaining about the performance of the UK’s Information Commissioner.^3^ Such complaints are not limited to the UK. In July 2020, the Irish High Court gave permission for a judicial review of the Data Protection Commissioner in respect of the delay dealing with complaints. It is to be noted that Ireland is the home to many tech companies’ European headquarters, and thus, these delays can impact more broadly upon EU citizens. However, equally, there are many examples of active engagement and investigation.

In terms of moving to cover new developments, the GDPR is not a prescriptive piece of legislation and, as such, its ‘vagueness by default’ is intended to ensure that the regulation maintains its relevance, allowing for application to new technologies, including FRT. Even more importantly, the GDPR holds some sway outside of the EU as well, since any business dealing with the bloc has to adhere to the rules when managing European’s data, even if those same rules do not apply in their own domestic jurisdiction. This is generally known as ‘The Brussels Effect’ [12, 13]. In practice, where FRT are rolled out in the EU, this means that it is much easier to hold FRT operators accountable, as there is no need to navigate a complex web of regional laws, and the operators themselves are more consistent in their behaviour, unable to use the splintering of regulation to their advantage. In addition, companies will often roll out the same systems globally, meaning that those outside the EU may benefit from some read over of standards. However, this is not to say that the systems will then be operated and managed in the same ways globally.

In terms of AI more specifically, this has become a focus for the EU and UK regulators and governments. The UK Information Commissioner’s Office (ICO) has recently published [14] guidance on AI auditing, supported by impact assessments. Although this guidance marks an important start towards specific guidance tailored towards the compliance of AI systems, we are still lacking case studies and dedicated frameworks to address this problem in a standardised way [15]. Recently, the EU has engaged with the need to actively manage the ethics and legislation that sit around AI innovation. A 2019 press release by the European Data Protection Supervisor Wiewiórowsk, called out the accountability and transparency concerns of facial recognition, particularly around the input data for facial recognition systems stating, “the deployment of this technology so far has been marked by obscurity. We basically do not know how data are used by those who collect it, who has access and to whom it is sent, how long do they keep it, how a profile is formed and who is responsible at the end for the automated decision-making.” [16]. As such, the European Commission began publishing a roadmap for dealing with AI. In April 2021, the European Commission released documentation on its approach to AI, which includes an aspiration to harmonise all legislation and bring in a specific Artificial Intelligence Act. FRT more specifically have yet to be dealt with in detail but, within the proposals for harmonisation, law enforcement systems are categorised as high risk. It is stated that AI systems used by law enforcement must ensure, “accuracy, reliability and transparency… to avoid adverse impacts, retain public trust and ensure accountability and effective redress” [17]. The documentation draws out areas of greater concern focusing on vulnerable people and those contexts where AI systems failures will have greater consequences. Examples include managing asylum seekers and ensuring individuals have a right to a fair trial. The importance of data quality and documentation is highlighted [17]. The Commission states that there must be oversight regarding:

“the quality of data sets used, technical documentation and record-keeping, transparency and the provision of information to users, human oversight, and robustness, accuracy and cybersecurity. Those requirements are necessary to effectively mitigate the risks for health, safety and fundamental rights…”

The place of the human in the system review is an important part of the process. In addition, the need for transparency is highlighted. However, what is not yet in place is a prescribed system for transparency and accountability. As the publications are currently at a high level, a need to drill down and consider case examples is necessary for delivery. There are some limitations to these publications and the recent publications by the EU have been criticized for not bringing in a moratorium on biometric technologies such as FRT [18]

In an EU context, in addition to the GDPR which dictates rules around managing personal data, privacy is further legislated for through the European Convention on Human Rights. As with the GDPR, this is enshrined in UK law as well as across all 27 EU Member States. The Human Rights legislation is potentially more holistic in terms of offering frameworks for consideration of law enforcement versus individual rights in the rollout considerations for FRT. It enshrines principles of equality and inclusion as well as privacy and rights to fair legal processes. The checks and balances of different and sometimes competing human rights are well established and tested through the courts. Under the terms of the law, individuals can bring legal cases, and, in the EU Member States (although not the UK), cases can progress to the European Court of Human Rights. However, there is not the same active regulatory framework sitting around the legislation which provides for quicker and cheaper routes to justice, and which can actively take action without the requirement for an individual to bring a case. Justice through the European Courts most normally is expensive, uncertain, and takes years. In addition, the requirements for accountability and design documentation for human rights compliance are not explicitly enshrined in the law. In terms of transparency, aspects of accountability for policy more generally fall under freedom of information legislation which is enacted at Member State level and differs very widely nation to nation in terms of public accountability requirements for administration more generally. There are also certain law enforcement and national security exemptions from freedom of information requirements. Finally, it is important to note that it does not bind on private entities who do not have the same accountability requirements.

In terms of actual FRT legal accountabilities, cases have been brought under both the GDPR and the Human Rights Act in respect of FRT. One such instance is the 2019 UK case of Bridges v. South Wales Police. Bridges, a civil rights campaigner, argued that the active FRT deployed by the police at public gatherings infringed on the right to respect for human life under the Human Rights Act 1998 and his privacy rights under the Data Protection Act 2018 (DPA 2018), the UK implementation of the GDPR. Relevant to this discussion, Bridges also claimed that, since the police failed to account for this infringement, its DPIA was not performed correctly [19]. After a lengthy litigation process, the court ruled in favour of Bridges, agreeing with the points above and additionally finding that the police had too broad a discretion regarding the use of FRT.

This example highlights the value of the GDPR (or similar legislative frameworks) and, in particular, the importance of the DPIA. Here, the impact assessment not only provided the basis for a large portion of the claimant’s argument, but it was also released to the public, making it easy for anyone with internet access to learn the details of the FRT data processing employed by the South Wales Police.^4^ In addition, the case shows that the DPIA is not a checkbox exercise but, instead, requires that the FRT operator possesses substantial knowledge about the inner workings of the algorithm and its wider repercussions.

The lawsuit also draws attention to the holistic understanding of privacy under the GDPR. In a country with less-developed data protection laws, it may be sufficient for an FRT operator to encrypt and anonymise faceprints, and, regardless of how they are collected, this will constitute sufficient protection; the GDPR goes to great lengths to ensure that this is never the case. Of particular importance are the concepts of PbD and privacy by default, as mentioned above and defined in Article 25 of the regulation. In this example, the South Wales Police ensured privacy by design, meaning that its facial recognition algorithms were built around data protection. That, however, was not enough, since the FRT were then deployed indiscriminately, which violated privacy by default—the amount of personal data collected was disproportionate with respect to the intended goal of identifying individuals on watchlists. As such, the police use of FRT for these processes had to be stopped. This “one strike and you’re out” approach to personal data collection goes a long way towards ensuring accountability in facial recognition, since it makes it much harder for the FRT operator to get away with negligent data processing for which there can be significant consequences. However, while the Human Rights legislation was deployed as part of the case, the lack of a published Human Rights Impact Assessment does diminish accountability in this regard. It is to be noted that a similar requirement to the provision of a DPIA, in regards to Human Rights Impact Assessments and human rights’ by design and default, could better improve citizen rights more generally.

In spite of the data protection legislation, it is important to highlight that authorities and corporate entities may fall short in their duties, which is why a proactive regulator is a significant attribute in the GDPR regime. In August 2018, upon the request of the London Mayor, the UK ICO started to investigate whether a private property company (Kings Cross Estate Services), which managed the area around Kings Cross, a critical London transport hub was using FRT in its CCTV. It emerged that for a number of years, this company had been using FRT for ‘public safety’ reasons, but had not properly disclosed or made people aware that the scheme was in operation. In addition, as part of this investigation it transpired that not only had it been using FRT to capture the images of all those people passing through the transport hub, but it had been working with the Metropolitan Police in London to check and match for certain people entering the area. A data sharing agreement was in place with the intention of providing for the potential identification of wanted individuals, known offenders, and missing persons. Over a 2-year period from 2016 to 2018, the Police passed images of seven people to the property entity. These people had been either arrested and charged, reprimanded, cautioned, or given a formal warning for offences. However, it was clear that the Police had failed to disclose that the scheme existed. [20]. That said, more generally the ICO has found that it is acceptable for the Police to use FRT and that there is a great deal of public support for its use, but that nevertheless it must be done so in a carefully targeted way taking into account individual’s Article 8 human rights to privacy [21].

Reflecting on the position of the Regulators and their investigatory powers, one of the most active national data protection bodies in the EU is the Swedish Authority for Privacy Protection (IMY), formerly known as the Swedish Data Protection Authority. In recent years, it has been involved in two FRT cases of note: a school using FRT to monitor class attendance [22], and the police using facial recognition software [23].

The first case, while not related to law enforcement, showcases how a data protection authority’s independence and legal expertise can ensure accountability where an individual or a civil organisation would not have been able to do so for various reasons. In this instance, the IMY “became aware through information in the media” that the school was trialing FRT on its students and decided to intervene. In the ensuing process, the authority found that the school’s use of facial recognition did not satisfy proportionality and necessity, which also led to the DPIA being conducted incorrectly. Most importantly, the IMY ruled that the consent that was given by the children’s parents to the school was invalid, as the students were in a position of dependence (school attendance is compulsory). The school’s board was subsequently fined approximately €20,000.

There are several important aspects to this example. First, note that the IMY intervened in the case on its own volition, without receiving any complaints or being asked to take action. This autonomy is important, as individuals may not always be able/willing to alert the authorities when their data are being collected and/or processed unlawfully. The reason why none of the parents came forward could be that they did not possess enough legal expertise to notice the problems in the FRT deployment or did not feel able to challenge the school given their own and their children’s relationship with it. The IMY had independence, sufficient knowledge, and a position of power to hold the school accountable. Finally, note the “one strike and you’re out” approach mentioned above. While the school made reasonable efforts to comply with the legal requirements—the faceprints were recorded on a hard drive connected to an offline computer locked away in a cupboard, and a DPIA was conducted—it failed to ensure *complete* compliance, and so was prosecuted.

The second example concerns the use of FRT by the Swedish police. The IMY found that the police failed to conduct a DPIA and were negligent enough to let unauthorised employees access the software, after which it imposed a fine of €250,000. Here, the law enforcement was ignorant to any negative consequences of FRT use and did not take appropriate active PbD steps; as a result, it was held accountable for its failings.

Exact data on how widespread FRT are across the EU is difficult to find, but the technologies are not ubiquitous yet. In 2019, 12 national police forces had already deployed facial recognition with 7 more planning or testing deployment at that date. Deployment has been deemed to be much slower than in USA [24]. This may in part be due to the fact that it is also surrounded by much more suitable, uniform legislation, greater transparency, and active data protection authorities—all of these components will play a large role in making Europe a better model for facial recognition accountability. However, in the context of FRT, it is important to note that a lot of the development has happened outside the boundaries of the EU and UK. As such, while the EU may have set a high bar in terms of requiring PbD, much FRT application happens within a USA context.

## The USA ethical and legislative landscape for FRT in a law enforcement context

Having considered the European regulatory framework, strongly positioned to ensure some forms of ethical considerations before the deployment of FRT, we now turn to a much more fragmented legislative territory: the United States of America (USA). Within USA, FRT are heavily used by law enforcement, affecting over 117 million adults [25], which is over a third of the country’s total population. FRT rollouts are widespread, yet an average citizen has very limited means of holding its operators accountable should it be misused. The USA was an early adopter of freedom of information laws, passing the federal Publication Information Act in 1966, with individual state laws being passed after this date. This set of legislation provides for state authorities to answer for their policies and actions on receipt of a freedom of information request. This does not impact on private companies who are not held accountable in the same way. In addition, there are certain exemptions under the legislation for law enforcement and national security purposes. There are some sector-specific privacy laws, covering, for instance children online, but no overarching data protection law akin to the GDPR. These federal laws are then enforced by the Federal Trade Commission, which has an extremely broad mandate of protecting consumers against deceptive practices; it is not comparable, however, to the data protection authorities in European countries [26]. Such a massive rollout of FRT without a regulator/ombudsman to investigate is a cause for concern as it then relies on individual legal action to call out wrongdoings. In addition, there are very considerable state-by-state differences, and a notable lack of requirements for transparency or calls for that transparency.

This reliance on individual action originates from USA lacking any federal (or state) data protection authority. This means that there is no body which would actively represent and protect citizens’ interests, while possessing the legal and regulatory powers of the state. Moreover, as we have seen, data protection authorities can intervene on behalf of the citizen and enforce decisions without initiating court proceedings; in the USA, this is not an option—any conflict regarding FRT and related personal data has to be heard in court, necessitating lengthy and costly court battles (which is why citizen representation is so important). As a result, individuals often have to seek legal support from non-profit organisations; those who fail to secure it may not be able to hold FRT operators or providers accountable at all.

The second issue is centered around state-by-state differences; it occurs thanks to an absence of a general federal privacy legislation, with state law often providing only very basic rights for holding FRT operators accountable. The extent of privacy laws in most states is limited to notifying an individual if their data have been stolen in a security breach [27]—hardly a consolation for someone who has been affected by unintentionally biased or malicious use of FRT. Relevant to our discussion, at the time of writing, there is only one state (Illinois) that has legislation allowing private individuals to sue and recover damages for improper usage and/or access to their biometric data, including faceprints [26]. However, even if you are lucky to live in Illinois, holding a malicious FRT provider or operator, private or public, accountable is likely to be difficult. Accountability relies on transparency—if, for instance, an individual would like to sue an FRT provider on the basis of a privacy violation, they will need some knowledge of how their data are processed. This is where the USA falls short; not only are the law enforcement and federal agencies notoriously secretive, but they often do not understand how their own FRT works in the first place. Without PbD and the requirements for a DPIA, there is less transparency on FRT processes, and it is harder to know exactly how processing is occurring and to hold operators to account. In addition, operators may often not have duly considered and weighted the implications of the FRT usage.

In a USA context, the law on privacy and use of FRT for localised law enforcement operates very much at a state-by-state level. Within this context, California is often held to be the state with the strongest privacy laws; in 2020, it strengthened its existing privacy laws with the California Privacy Rights Act (CCPA), which established the California Privacy Protection Agency and extended residents’ rights in terms of how business could collect and use their data. However, notably, it did not touch on any privacy powers in respect of law enforcement, and, in tandem with the CCPA, the state started to try to introduce a Facial Recognition Bill to enhance the use of FRT for law enforcement purposes. It is to be noted that some cities in California (e.g., Berkeley and San Francisco) have banned FRT usage. Interestingly, the Bill received lobbying support from Microsoft, but was fiercely campaigned against by Civil Rights groups, and as such, it was not passed in June 2020. This period marked a growing sense of unease with the ethics around FRT. In the same month, IBM stated that it would cease all export sales of FRT. In its statement, it described FRT as akin to other innovations such as nuclear arms on which the USA has had to seize a lead for the protection of its citizens [28]. In addition, it highlighted the flaws in the technology, for example its failure to deal with Black and Asian faces with sufficient accuracy. At the same time, another big tech entity, Amazon stated that it would cease to sell FRT to the Police for 1 year to give Congress time to put in place new regulations to govern its ethical usage. Microsoft followed suit stating, “we will not sell facial recognition technology to police departments in the United States until we have a national law in place, grounded in human rights, that will govern this technology" [29]. Each of these entities clearly became concerned about the potential misuse of the technology by law enforcement agencies which IBM said had caused concerns since the revelations by Edward Snowden in 2014 [29]. Clearly, there were valid ethical concerns about the development of FRT. However, when beneficial influences leave the marketplace, this may open up the field to less ethical developers. Each of these entities has a process for reviewing the ethics of technology roll outs, for example, IBM has an Ethics AI Board led by a Chief Privacy Officer. It is difficult to know how ethical or effective these private entities are where there is such limited transparency, although clearly these large global corporations worry about their images. This was evidenced in the case of Google which received international press attention and criticism when it fired Timnit Gebru, co-lead of its Ethical AI Research Team, for refusing to edit out certain statements from a research article on AI [30], and as a result of the controversy, it has since had to change its publication approach.

The concerns of private enterprise and the relationship with law enforcement and national security have been recognised at a national level. For example in the context of the Federal Bureau of Investigation (FBI), there have been hearings in Washington on the acceptable use of FRT.^5^ At this hearing, it was stated that the “FBI has limited information on the accuracy of its face recognition technology capabilities.” These hearings called for greater accountability and transparency in the use of the technologies, although definitive outcomes from the hearings are still awaited.

A recent illustration of the current opacity of the USA system is demonstrated in the case of Willie Allen Lynch, a black man convicted in 2016 by a Florida court of selling cocaine; the Police Department made the decision to arrest him based on a facial recognition match, among other factors. In an attempt to appeal the decision, Lynch argued that the facial recognition system made an erroneous match (a reasonable statement, given FRT’s known inaccuracy with black faceprints [9]), proving this, however, required the police to turn over the photo in question and the list of possible faceprint matches offered by the system, which it refused to do. Strikingly, the detectives involved in the case admitted that, while the FRT rated Lynch’s faceprint as the closest match, they did not actually know how the rating system worked or even which scale the rating was assigned on. Ultimately, the court ruled in favour of the Police Department, and Lynch was never given access to the photo and potential matches [31].

On a federal level, the issues of a lack of transparency and accountability persist; an attempt by the American Civil Liberties Union (ACLU) to gather information about the use of FRT by the Department of Justice, the FBI and the Drug Enforcement Administration failed, since none of the agencies responded to a Freedom of Information Act request. Undeterred, the ACLU pursued legal action, with results yet to be seen—there has been no information about the case since October 2019, when the initial complaint was filed [32]. In addition, the ACLU has called out the Government’s and private enterprises’ surveillance operations at airports and customs boundaries across the USA [33].

In regard to private companies, as previously noted, these are not caught by freedom of information laws and can often afford legal firepower beyond the reach of even the wealthiest individuals. Clearview AI, one of the leading providers of FRT to the USA law enforcement agencies, supplies the technologies to more than 600 police departments across USA [34]; the ACLU filed a lawsuit against the company in the state of Illinois, arguing that it collected faceprints without consent, as required by the state’s Biometric Information Privacy Act [35]. Filed in May 2020, the case remains active at the time of writing, accumulating a seemingly endless stream of motions, memoranda, and briefs from both sides. The amount and complexity of the legal paperwork on a case that has not even been heard yet is illustrative of how fiercely opposed the company is to any efforts to hold it accountable, and it is problematic for ordinary citizens to follow the lawsuit through on their own; although crowdsourcing and group action has become a reality for legal cases, as seen in the actions brought by the Austrian Max Schrems in the EU. In addition, there has been a class action brought against the Department Store Macy’s in Illinois for its use of FRT [36], so such legal action may become more common. Nevertheless, a mature democratic nation should have other solutions in place.

This absence of the threat of litigation removes the proverbial sword hanging above the FRT providers’ heads, allowing them to have a free-for-all feast on user information. For instance, Clearview AI openly discloses information about scraping Facebook user profiles for images to build up its reference database [34], even though this action is explicitly prohibited by the website’s terms of service. IBM, in a similar fashion, collected individuals’ Flickr photos without consent; the affected users were not given a feasible way of deleting their information from the database [37]. A complete absence of data protection and privacy rights is hugely problematic.

## Conclusion and recommendations

FRT is no longer a topic of science fiction or a concern for the future. It is here now, impacting people’s lives on a daily basis, from wrongful arrests to privacy invasions and human rights infringements. The widespread adoption of this technology without appropriate considerations could have catastrophic outcomes, and ultimately may jeopardise its development if some jurisdictions decide to ban the use of the technology for an indefinite amount of time [38]. However, critical in the success of FRT is the transparency and accountability in each stage of its development and usage and the ability to audit and challenge as required. The idea of power is particularly linked to the intended, and actual, outcomes of FRT, which should not be dissociated from discussions around accountability.

This discussions in this article makes the case that at all stages of the FRT process in all aspects of design and use including specific contexts, there is a requirement to document and account for the usage ensuring mechanisms for transparency and challenge. The GDPR provides a good regulatory starting point to address some of its concerns. However, the ethical considerations of this technology go far beyond issues of privacy and transparency alone. It requires broader considerations of equality, diversity, and inclusion as well as human rights issues more generally. As such other forms of assessments, such as Human Rights Impact Assessments, in addition to DPIA, should be part of the development and rollout of FRT—a DPIA alone is insufficient. These Assessments should be automatically required to be put into the public domain. In addition, the requirements must equally be enacted upon both public and private enterprises with transparency and accountability requirements. In conjunction with these steps, global regulators are needed with powers to actively investigate each aspect of the development and deployment processes of FRT in case contexts, and with powers to step in, stop and fine inappropriate FRT development and deployment. In addition, there should be more normal audit processes required for FRT deployment just as there are for financial oversights. The societal impacts for FRT misconduct are not to be underestimated.

We conclude this paper with the recommendation of ten critical ethical questions that need to be considered, researched, and answered in granular detail for law enforcement purposes and which in addition have read over to other AI development. It is suggested that these need to be dealt with and regulated for. The questions are:

1. Who should control the development, purchase, and testing of FRT systems ensuring the proper management and processes to challenge bias?
2. For what purposes and in what contexts is it acceptable to use FRT to capture individuals’ images?
3. What specific consents, notices and checks and balances should be in place for fairness and transparency for these purposes?
4. On what basis should facial data banks be built and used in relation to which purposes?
5. What specific consents, notices and checks and balances should be in place for fairness and transparency for data bank accrual and use and what should not be allowable in terms of data scraping, etc.?
6. What are the limitations of FRT performance capabilities for different purposes taking into consideration the design context?
7. What accountability should be in place for different usages?
8. How can this accountability be explicitly exercised, explained and audited for, for a range of stakeholder needs?
9. How are complaint and challenge processes enabled and afforded to all?
10. Can counter-AI initiatives be conducted to challenge and test law enforcement and audit systems?

We are at a tipping point in the relationships and power structures in place between citizens and law enforcers. We cannot wait to step in and act, and in fact, there are many potential solutions to better ensure ethical FRT deployment. However, this is currently an ethical emergency requiring urgent global attention.

## Footnotes

1 For example, see McGregor, L. (2018) ‘Accountability for Governance Choices in Artificial Intelligence: Afterword to Eyal Benvenisti’s Foreword’, *European Journal of International Law*, 29(4), pp. 1079–1085.; Shah, H. (2018) ‘Algorithmic accountability’, *Philosophical Transactions of the Royal Society A: Mathematical, Physical and Engineering Sciences*, 376(2128), p. 20,170,362. https://doi.org/10.1098/rsta.2017.0362; Buhmann, A., Paßmann, J. and Fieseler, C. (2020) ‘Managing Algorithmic Accountability: Balancing Reputational Concerns, Engagement Strategies, and the Potential of Rational Discourse’, Journal of Business Ethics, 163(2), pp. 265–280. https://doi.org/10.1007/s10551-019-04226-4.0.

2 For the formal definition of the DPIA, see GDPR Article 35.

3 See https://www.openrightsgroup.org/app/uploads/2020/08/Letter-for-MPs-Final-sigs-1.pdf.

4 This particular assessment is available here: https://afr.south-wales.police.uk/wp-content/uploads/2019/10/DPIA-V5.4-Live.pdf.

5 See for example the 2019 report at https://oversight.house.gov/legislation/hearings/facial-recognition-technology-part-ii-ensuring-transparency-in-government-use.
